# Following Eye Gaze Activates a Patch in the Posterior Temporal Cortex That Is not Part of the Human “Face Patch” System

**DOI:** 10.1523/ENEURO.0317-16.2017

**Published:** 2017-03-23

**Authors:** Kira Marquardt, Hamidreza Ramezanpour, Peter W. Dicke, Peter Thier

**Affiliations:** 1 Department of Cognitive Neurology, Hertie Institute for Clinical Brain Research, 72076 Tübingen, Germany; 2 Graduate School of Neural and Behavioural Sciences, University of Tübingen, 72074 Tübingen, Germany; 3 International Max Planck Research School for Cognitive and Systems Neuroscience, University of Tübingen, 72074 Tübingen, Germany; 4 Werner Reichardt Centre for Integrative Neuroscience (CIN), University of Tübingen, 72076 Tübingen, Germany

**Keywords:** face patch, gaze-following patch, joint attention, posterior superior temporal sulcus

## Abstract

Humans follow another person’s eye gaze to objects of interest to the other, thereby establishing joint attention, a first step toward developing a theory of the other’s mind. Previous functional MRI studies agree that a “gaze-following patch” (GFP) of cortex close to the posterior superior temporal sulcus (STS) is specifically implicated in eye gaze-following. The location of the GFP is in the vicinity of the posterior members of the core face-processing system that consists of distinct patches in ventral visual cortex, the STS, and frontal cortex, also involved in processing information on the eyes. To test whether the GFP might correspond to one of the posterior face patches, we compared the pattern of blood oxygenation level–dependent (BOLD) imaging contrasts reflecting the passive vision of static faces with the one evoked by shifts of attention guided by the eye gaze of others. The viewing of static faces revealed the face patch system. On the other hand, eye gaze-following activated a cortical patch (the GFP) with its activation maximum separated by more than 24 mm in the right and 19 mm in the left hemisphere from the nearest face patch, the STS face area (FA). This segregation supports a distinct function of the GFP, different from the elementary processing of facial information.

## Significance Statement

Human observers follow another person’s eye gaze to objects and locations of interest to the other, thereby establishing joint attention, a major step toward developing a theory of the other’s mind. Previous functional MRI (fMRI) studies agree that a patch of cortex around the posterior superior temporal sulcus is specifically implicated in eye gaze-following. This gaze-following patch is located in the same region as the posterior elements of the face patch system, also extracting information on the eyes. Using fMRI, we show that the gaze-following patch is distinct from the face patch system, supporting a role beyond the elementary processing of facial information accommodated by the face patch system.

## Introduction

Eye gaze, head, shoulder, and trunk orientation are important examples of body cues that offer compelling information on the object and location of interest to the other, drawing the observer’s attention to the same object and location, thereby establishing “joint attention,” a first and major step toward developing a theory of the other’s mind (Baron-Cohen, 1994, 1995; Emery, 2000; Langton and Bruce, 2000; Shimojo et al., 2003). In humans, eye gaze is arguably the most important social cue guiding the observer’s attention (Emery, 2000). A precise localization of the relevant machinery has recently been provided by a number of functional MRI (fMRI) studies. This work has identified a circumscribed region in the posterior superior temporal sulcus (pSTS) of both hemispheres, adjacent to the middle and superior temporal gyri, often referred to as pSTS region or area or, more loosely, the gaze-following patch (GFP; Puce et al., 1998; Allison et al., 2000; Hoffman and Haxby, 2000; Pelphrey et al., 2003, 2004; Materna et al., 2008a; Laube et al., 2011). A cortical area involved in macaque monkeys’ head gaze-following, the monkey GFP, has recently been described in a comparable cortical region that may eventually turn out to be homologous with the human GFP in the pSTS (Marciniak et al., 2014).

The extraction of eye gaze orientation requires knowledge of the orientation of the eyes relative to the face and ultimately also knowledge about the orientation of the other’s face relative to the observer and the world. This need to care about particular aspects of faces might suggest that eye gaze-following may build on information provided by the parts of cortex known to be devoted to the processing of faces, including their constitutive elements such as the eyes. Actually, this influence of the eyes is suggested by a number of studies that have demonstrated that, for instance, information on identity and emotional expression, provided by the eye region, influences not only perception but also the activity in distinct face patches (Fox and Damjanovic, 2006; Chan and Downing, 2011).

In fact, the human GFP, lighting up in gaze-perception tasks, is located in close vicinity to face-selective areas in the ventral visual cortex. This raises the possibility that the GFP may actually be one of the members of this face-processing network that involves distinct elements in the ventral visual cortex and frontal cortex, namely the occipital face area (OFA), the fusiform face area (FFA), the STS face area (STS-FA), and the inferior frontal gyrus face area (IFG-FA; Kanwisher et al., 1997; Haxby et al., 2000; Tsao et al., 2008). These areas are interconnected and seem to be devoted to particular aspects of faces. For instance, the FFA emphasizes the encoding of constant aspects of the face underlying identity decisions (Grill-Spector et al., 2004). On the other hand, the STS-FA, the face-selective area closest to the known location of the GFP, has been shown to contribute to encoding changeable aspects of faces such as facial expression and face orientation, the latter an aspect obviously important for gaze-following (Puce et al., 1998; Wicker et al., 1998). Could it be that the STS-FA is actually part of the machinery for gaze-following, rather than being confined to providing information on face orientation? In this case, we would expect at least partial overlap between the GFP and the STS-FA. In view of the interindividual variability in the location of the GFP and also the STS-FA, the question whether the two overlap requires testing the same subjects in gaze-following and face-perception tasks. Using well-controlled fMRI paradigms in the same set of subjects, we show that the two systems are actually well segregated, a finding that clearly indicates that the GFP accommodates a functionality not found in the face-selective areas, although most probably building on pertinent information contributed by the latter.

## Material and Methods

### Subjects and instrumentation

Eleven adult male and nine adult female subjects, age range 21–46 years (mean 26, SEM 5.5 years) participated in the current study. All participants were right-handed and healthy and had normal or corrected-to-normal vision. Subjects were provided with transparent and comprehensible information about the study goals and the procedures involved and gave their written consent. Participants ran a training behavioral session before an imaging session to minimize errors inside the MRI scanner caused by potential misunderstanding of task rules or a lack of practice. The study was approved by the Ethics Review Board of Tübingen Medical School and was conducted in accordance with the principles of the 1964 Declaration of Helsinki.

In the training session, subjects’ eye movements were recorded deploying a commercial Eye Tracker (Chronos Vision C-ETD). During the imaging session, subjects’ heads were stabilized by foam rubber to minimize residual head movements. The visual stimuli (32° × 24° visual angle) were presented on a translucent screen using an LCD projector (NEC GT 950, 1024 × 768 pixels) viewed by the subjects via a two-mirror system with 60-cm distance between the translucent screen and the subjects’ eyes. During the imaging procedure, a certified, MRI-compatible Eye Tracker (SMI iView X MRI-LR) was used to record the subjects’ eye movements. The recorded eye movements were evaluated offline after the experiments.

### Visual stimuli and experimental tasks

The participants had to perform three tasks. The first task required the observer to extract the portrait’s eye-gaze direction and make a saccade toward one of a set of five spatial targets that the portrayed demonstrator looked at (gaze-following task). The second task also required an indicative saccade to targets singled out by information provided by the same demonstrator portraits; however, in contrast to the first task, a different rule applied. Rather than following the demonstrator’s gaze, the observer was required to make a saccade to the target that had the same color as the portrayed demonstrator’s iris (color-matching task). Note that the visual information provided in the two tasks was the same, i.e., in both tasks the iris color varied from trial to trial, adopting the distinctive color of one of the five targets, arranged on a horizontal line met by the demonstrator’s gaze axis. Using the same visual stimuli for the gaze-following task and the control task and requiring the same behavioral responses, any differences in the associated blood oxygenation level–dependent (BOLD) imaging responses would have to be caused by differences between the cognitive operations induced by the two sets of cues. Finally, participants were subjected to a third experiment that required fixation on a small dot while passively viewing images of faces and nonface stimuli, centered on the fixation dot, not requiring any behavioral response (passive face perception task).

The portraits used in the gaze-following/color-matching tasks (collectively referred to as the “active tasks”) were photographs of a female in front of a white background. She was looking either straight into the camera (baseline fixation picture) or to one of five dot targets arranged on a horizontal board, 25° below the straight-ahead axis in the fronto-orthogonal plane, with a visual angle of 12.5° between targets. The digital photographs were processed using Adobe Photoshop CS5 to replace the original background with a black-and-white random dot pattern and color the portrait’s iris and the five targets with five different colors (dark blue, light blue, green, light brown, and dark brown).

The tasks were run in separate blocks. Each block started with a written task instruction on the projection screen (either gaze-following or color-matching) present for 5 s. The whole block lasted for 95 s and contained 10 trials. Each trial started with a baseline fixation picture with direct gaze (lasting for 5 s), immediately followed by one of five possible portraits (target portraits), present for 4 s, with the demonstrator’s gaze directed at a specific target and exhibiting a distinct iris color. Within one block, these 10 trials were sorted randomly. The whole experiment contained four sessions, each involving two blocks of gaze-following and two of color-matching, one after another.

**Fig. 1.**
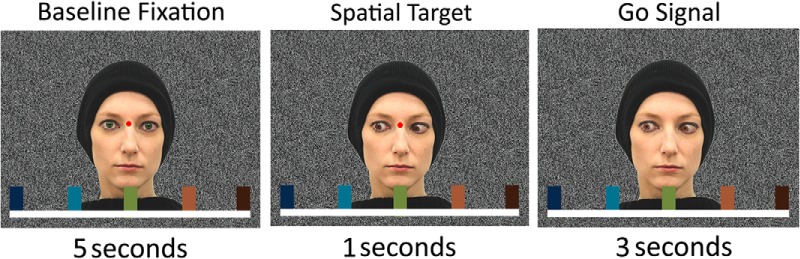
Sequence of visual stimuli in the active task. At the beginning of each block of trials, a written instruction (either gaze-following or color-matching) was presented on the screen for 5 s. Each trial started with a baseline fixation picture with direct gaze (lasting for 5 s), immediately followed by one of five possible portraits (target portraits), present for 4 s, with the demonstrator’s gaze directed at a specific target and exhibiting a distinct iris color. Subjects were not allowed to make an eye movement until the disappearance of the fixation target. Afterward, alternately 10 fixations (each 5-s duration) and 10 trial pictures (each 4-s duration) were presented. The demonstrator has agreed for her portrait to be published.

**Fig. 2.**
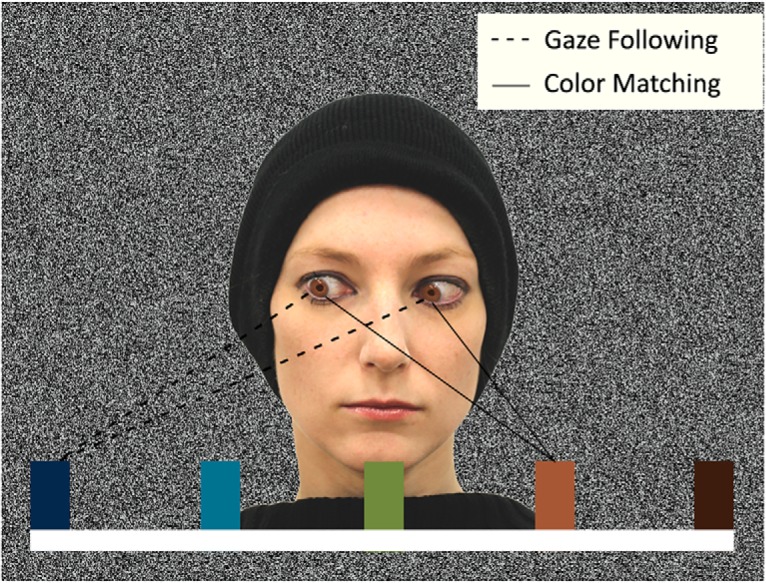
Illustration of the first experiment’s stimulus. The eyes of the person are directed to the dark-blue target (gaze cue), but the person’s iris color corresponds to the light-brown target (color cue). According to the introduced condition at the beginning of the block, the subject would have to make a saccade toward the dark-blue target (gaze-following condition) or toward the light-brown target (color-matching condition). The demonstrator has agreed for her portrait to be published.

During the presentation of the baseline fixation picture, the subjects were asked to fixate on a small dot with 0.3° visual angle radius presented between the demonstrator’s eyes oriented straight ahead. This fixation dot was also present in the target portraits for the first 1 s and then turned off. The disappearance of the fixation point served as the “GO” signal for the participants to perform their saccade to the target singled out by the prevailing rule (gaze-following vs. color-matching). The subjects had to stay with their eye-gaze on the chosen target until the baseline fixation picture, now serving as “GO” signal, appeared again (see Fig. 1 and Fig. 2). Implementing this “GO” signal seemed to be necessary to allow us to reveal differences in BOLD signals between gaze-following and color-matching. Otherwise, possibly dominating BOLD signals associated with undelayed saccades might have concealed the differential BOLD activity associated with the preceding processes.

The stimuli deployed in the passive face perception task (in short, “passive task”) were photographs of human faces (females and males), hands, and bodies plus manmade objects of daily life as well as food, each subtending a 12° visual angle. Facial stimuli were taken from the Radboud Face Database (Langner et al., 2010), showing females and males with averted gazes. Adobe Photoshop CS5 was used to create scrambled versions of all photographs and replace the backgrounds with the same black-and-white random dot background used in the gaze-following paradigm. Stimuli were presented in four sessions, each containing 10 blocks of 16 photographs. The sequence of blocks was the same in each session, but photographs were randomly distributed within a block. Each block lasted 38 s and started with the presentation of a fixation dot in front of a black-and-white random dot background for 5 s, followed by 16 photographs (presentation time, 1 s each) with black screens present for 0.2 s in between. During presentation, subjects were asked to maintain fixation on a small dot in the middle of the screen while viewing the photographs.

### MRI imaging and preprocessing

A 3 Tesla MR-Scanner (Siemens Magnetom Trio Tim syngo MR B17) was used to scan subjects’ brains. We used a T2^*^-weighted echo-planar sequence (TE, 35 ms, TR, 3000 ms; flip angle, 90°) covering the whole brain (44 transverse slices; matrix 64 × 64; slice thickness, 2.5 mm; in-plane resolution, 3 × 3) for image acquisition during the experiments. We used a T1-weighted, magnetization-prepared, rapid-acquisition gradient-echo sequence (MP-RAGE with TE, 2.92 ms; TR, 2300 ms; TI, 1100; flip angle, 8°; 176 × 256 × 256 voxel; voxel size, 1.0 × 1.0 × 1.0 mm) for the structural, anatomic scans. A total of 945 images were taken from each subject.

The preprocessing and analysis of the images was done with the statistical parametric mapping program package SPM8 (Wellcome Department of Cognitive Neurology, London, UK, http://www.fil.ion.ucl.ac.uk/spm) running on Matlab 2013. Images of each subject were reoriented by setting the origin to the anterior commissure and correcting for slice time (number of slices, 44; TR, 3 s; TA, 2.93; slice order, interleaved descending; reference slice, 22). Functional scans were spatially realigned (registered to first and mean images resliced). The anatomic scan was coregistered to the mean volume of the functional images and was normalized to the Montreal Neurologic Institute space (Friston et al., 1995). Functional images were normalized to the anatomic scan and then smoothed using a 7-mm full-width half-maximum Gaussian filter. Time series in each voxel were high pass–filtered with a cutoff frequency of 1/128 Hz.

### MRI data analysis

To estimate the BOLD activation patterns associated with the experimental tasks, we assumed a standard hemodynamic response function, reflecting the task variables according to a general linear model (GLM). In the active task, the onset of the portrait defined time 0 of the ensuing event trace. We distinguished three different event types: fixation, gaze-following, and color-matching. In the passive task, the appearance of the first image in each block determined time 0 of an event trace spreading across the whole block.

The estimated head movements of the subjects during the sessions were considered as regressors of no interest in the GLM in addition to covariates of interest (experimental conditions: fixation, gaze-following, color-matching, faces, and nonfaces). For the active tasks, the following contrasts were calculated for each subject: response to gaze-following and color-matching versus baseline fixation and response to gaze-following versus color-matching and vice versa. For the passive task, contrasts between responses to faces and all nonface stimuli including the scrambled faces were calculated. *t*-statistics were used to identify significant changes (*p* < 0.0001 for the active task and a more conservative threshold of *p* < 0.001 for the passive task, taking into account its lower statistical power) in the BOLD signal at the level of individual subjects. To test whether results obtained for individual subjects are valid at the population level, we conducted a second-level analysis, deploying a random-effects model, comparing the average activation for a given voxel with the variability of that activation over the examined population (Friston et al., 1999). The average activation for a given voxel was taken as significant if the probability *p* provided by *t*-statistics fell below 0.0001 (uncorrected) for that voxel and in at least six neighboring ones. To optimally visualize and measure the cortical representations, statistical *t*-maps were projected onto inflated and flattened reconstructions of cortical surface gray matter using Caret (http://brainvis.wustl.edu/wiki/index.php/caret).

## Results

### Behavioral findings

**Fig. 3.**
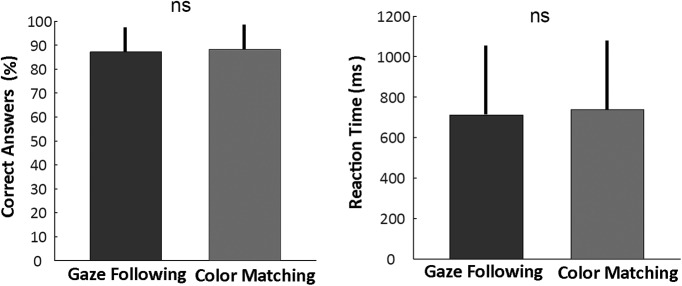
Behavioral data for gaze-following (dark gray) and color-matching (light gray) showing no significant difference in either mean accuracy or mean reaction time (time between the go signal and the start of the eye movement (*n* = 20 sessions, 160 correct trials). Error bars represent SE.

In the active experiment, participants were instructed to identify the target either by following the portrait’s eye-gaze (gaze-following) or, alternatively, to identify it based on a color match with the iris of the portrayed demonstrator and execute a saccade to the target. In the first case, eye color, and in the second case, eye gaze direction, had to be discounted. The two variants of the active task did not differ with respect to the visual information available or the oculomotor behavior prompted but with regard to the cognitive strategy required to solve the task. One might argue that the two different strategies to be pursued might have been associated with different levels of difficulty and, consecutively, also different subjective task loads. This did not seem to be the case, as task performance was very similar. Participants performed the task in the scanner with high accuracy well above chance level (20%) in the gaze-following condition (correct responses: mean, 87%; SEM, 11%) as well as in the color-matching condition (correct responses: mean, 88%; SEM, 11%). Kolmogorov–Smirnov test showed that reaction times and correct responses showed a normal distribution. A paired *t*-test showed no significant difference in the number of correct responses (*p* = 0.61) or reaction times (*p* = 0.32) between the two conditions (gaze-following reaction time: mean, 711 ms; SEM, 366 ms; color-matching reaction time: mean, 736 ms; SEM, 341 ms; Fig. 3).

### BOLD responses to gaze-following and color-matching

**Fig. 4.**
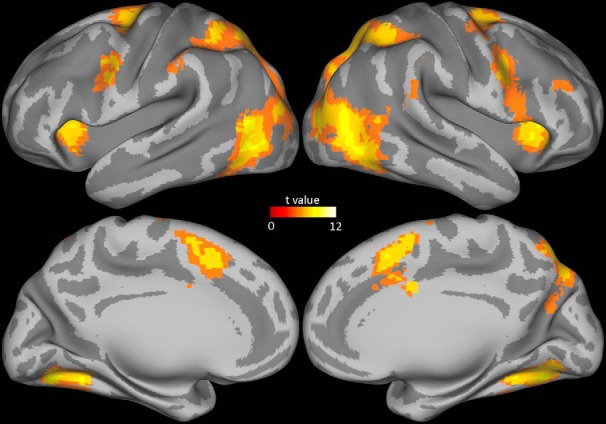
MRI group data showing the BOLD response for the contrast gaze-following versus baseline fixation.

**Fig. 5.**
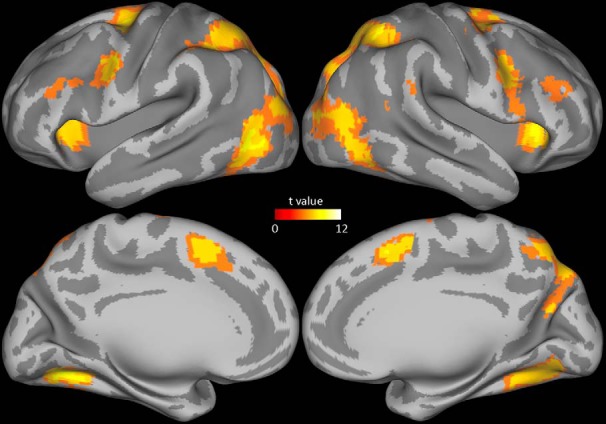
MRI group data showing the BOLD response for the contrast color-matching versus baseline fixation.

To identify brain areas activated during gaze-following, we looked at the contrast of gaze-following versus baseline fixation in a second-level analysis of the group data. This comparison delineated several brain areas in both hemispheres that had a significantly higher BOLD signal (*p* < 0.0001, in a cluster of six connected voxels each; see Fig. 4), among them dorsolateral prefrontal cortex, premotor cortex, supplementary motor area, cuneus, precuneus, fusiform gyrus, posterior middle temporal gyrus, inferior temporal gyrus, middle occipital gyrus, clustrom, middle frontal gyrus, inferior parietal lobule, superior parietal lobule, supramarginal gyrus, precentral gyrus, cingulate gyrus, superior frontal gyrus, lingual gyrus, superior occipital gyrus, parahippocampal gyrus, and cerebellum. This pattern was very similar to the one obtained when calculating the color-matching versus baseline fixation contrast (Fig. 5). The close, qualitative match between the patterns associated with the two tasks is not unexpected, given that both require the extraction of specific cues from faces to localize distinct objects to shift one’s attention to them.

**Fig. 6.**
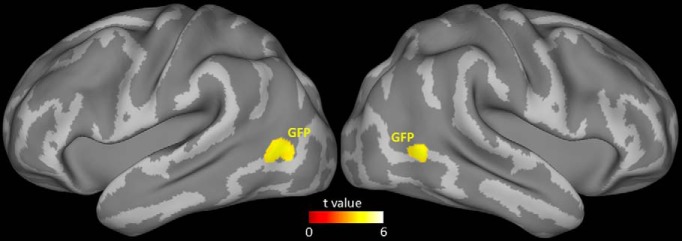
MRI group data showing the BOLD response for the contrast gaze-following versus color-matching. Activation maximum in right hemisphere in Talaraich coordinates (50, –64, 2).

To identify cortical regions specifically or more strongly activated by the need to exploit gaze direction, we calculated the BOLD contrast between gaze-following and color-matching. A significant contrast (statistical criteria as before) was found in a patch of cortex bilaterally in the posterior part of the middle and inferior temporal gyrus, specifically with the peak contrast at Talaraich coordinates right (50, –64, 2) and left (–54, –67, 6; see Fig. 6). This location of activity is similar to gaze-following– and gaze-processing–related activity found in previous fMRI studies (Hoffman and Haxby, 2000; Hooker et al., 2003; Pelphrey et al., 2005b; Materna et al., 2008a). We refer to the activated patch as the gaze-following patch (GFP) and the cortical region in which it is located as the pSTS.

### BOLD responses to the passive vision of faces

We identified cortex activated by the passive vision of static faces by delineating regions for which the contrast faces versus nonface objects (biological as well as nonbiological objects and scrambled faces were pooled) was significant in the second-level analysis (*p* < 0.001, uncorrected, six connected voxels). In accordance with previous studies (Ishai et al., 2005; Gobbini and Haxby, 2006; Fox et al., 2009), we found significant BOLD contrasts in the mid-fusiform gyrus bilaterally (these voxels are the FFA), the right inferior occipital gyrus (these voxels form the OFA), the posterior superior temporal sulcus bilaterally (these voxels correspond to the STS-FA), and the right inferior frontal gyrus (these voxels make up the IFG-FA). The highest BOLD contrast to faces was identified in the functionally defined STS-FA, located at Talaraich coordinates right (51, –42, 12) and left (–57, –48, 8). After identifying the face-selective regions in the second-level analysis, the BOLD time series underwent spatial smoothing with an 8-mm FWHM Gaussian blur, and the clusters of face-selective regions were extracted as a mask mapped on the cortical surfaces to assess their spatial relationship to the GFP later on.

### The pSTS gaze-following patch and the face patch are segregated

**Fig. 7.**
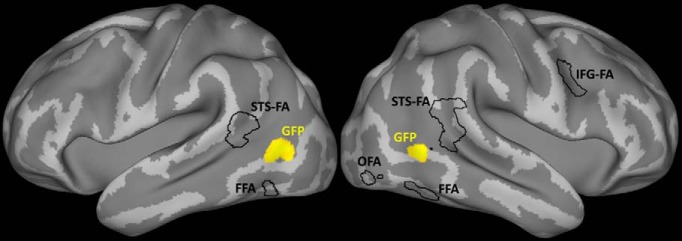
Spatial organization of face-selective areas and the gaze-following patch.

**Fig. 8.**
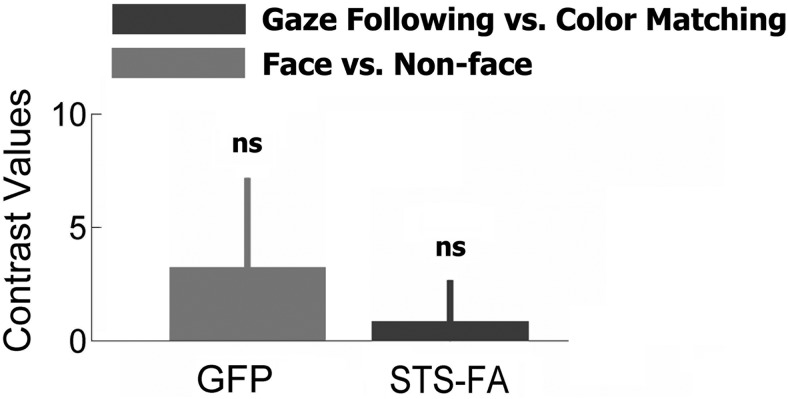
Selectivity of the individually defined STS-FA to gaze-following in contrast to the selectivity of the GFP to static face perception. Error bars indicate 90% confidence intervals. In the right STS-FA (Talaraich coordinates of the peak: 51, –42, 12), the mean contrast value for gaze-following is not significantly different from zero, in accordance with the assumption of a lack of gaze-following selectivity (*t*-test, *p* = 0.49). On the other hand, the contrast value for face perception in the right GFP (Talaraich coordinates of the peak: 50, –64, 2) is not significantly different from zero, meaning no face-selectivity (*t*-test, *p* = 0.20).

The fact that the GFP and the STS-FA, exhibiting the strongest BOLD contrast, were found in the same posterior part of the STS suggested that the two might overlap or, eventually, be even fully congruent. To investigate this possibility, we projected the two GFP and the face patches, including the one in the pSTS region, onto an inflated 3D representation of cortical surfaces using the PALS-B12 atlas of human cerebral cortex (Van Essen, 2005). This rendering did not exhibit any indication of overlap between the gaze-following patch and any of the face-selective regions. Actually, the boundaries of the GFP and the ones of the nearest STS-FA were separated by a gap of 4 mm (Fig. 7). We next defined the GFP and the STS-FA as our regions of interest (spheres with the diameter of 5 mm centered on the coordinates of the peak activities in these two areas in each individual subject to compare the response levels as captured by the contrast values for passive perception of static faces and gaze-following. As shown in Fig. 8, the average contrast values in the GFP for the passive face perception task did not differ significantly from zero (*t* test, *p* = 0.20), meaning that there was no selectivity for faces. Likewise, the mean contrast values in the STS-FA during gaze-following did not differ significantly from zero (*t* test, *p* = 0.49), correspondingly expressing a lack of selectivity to gaze-following. Hence, we may conclude that the GFP and the STS-FA are neighboring, yet nonoverlapping, areas with different functions. In six of 20 subjects, we could not delineate a significantly activated GFP and STS-FA at the level of the individual. Hence, these six subjects had to be excluded from a comparison of gaze-following–related activity with activity in individual delineated STS-FA and vice versa; i.e., face-selectivity test in the GFP. We also resorted to a conjunction analysis as an alternative to a random-effect analysis (Heller et al., 2007). This approach allows the assessment of how many subjects exhibit selective activations in each voxel and therefore shows the extent of overlap between gaze-following–related activity and activity evoked by static faces within and across subjects. This analysis did not show any overlap in individual subjects, passing the significance threshold of *p* < 0.001 (uncorrected).

## Discussion

With two separate fMRI experiments, performed on the same subjects, we tried to map the cortical areas underlying gaze-following and the establishment of joint attention and/or the passive perception of static faces. The two experiments were run on the same subjects to find out whether the cortical structures involved overlap. In the first experiment, consisting of two tasks, subjects were asked to either follow the eye gaze direction of portrayed demonstrators toward distinct spatial targets or, alternatively, to shift attention to the target whose color corresponded to that of the demonstrator’s iris. In accordance with previous work (Materna et al., 2008a), we found that a GFP lighted up bilaterally in the posterior part of the middle temporal gyrus when the BOLD signal evoked by eye gaze-following was contrasted with the BOLD signal evoked in the color-matching condition. Assuming that this contrast is able to eliminate activity due to visual stimulation or the indicative saccades required in both tasks, we may conclude that the neuronal machinery in the gaze-following patch in the pSTS might be responsible for the calculations needed to shift the observer’s attention based on eye gaze. Unlike the shifts of attention evoked by our color-matching paradigm, gaze-following is reflexive (Friesen and Kingstone, 1998). However, this does not mean that it would not be subject to cognitive control. Indeed, careful psychophysical experiments on monkey head gaze-following (Marciniak et al., 2015), probably homologous to human gaze-following, clearly show that with the exception of a small early reflex component, a substantial part of the gaze-following response can be suppressed. Hence, we can be confident that the BOLD contrast used to identify the GFP reflects differences in gaze-following–related processing and its cognitive control. Our paradigm vetoed an immediate behavioral response to the gaze cue as subjects had to delay the response until the occurrence of the “GO” signal. Hence, one might be concerned that the GFP activity we observed in this experiment might differ from the normal pattern evoked by spontaneous gaze-following. However, the spatial coordinates of the GFP identified here are in accordance with our previous findings on activations evoked by spontaneous gaze-following (Materna et al., 2008a).

In the second experiment, we used a classical static face localizer to map the face-selective regions potentially involved in extracting information on face and eye gaze orientation to clarify the anatomic relationship between the GFP and the members of this face patch system. In fact, we did not observe any overlap between the GFP and any of the face patches, in particular not with a patch in the posterior STS (STS-FA), which in view of its localization as described by previous work (Kanwisher et al., 1997; Haxby et al., 2000) might have been expected to overlap with the GFP. One might argue that a lack of overlap between the two is not surprising, given that the GFP is orchestrating shifts of attention guided by the eyes, i.e., just one of many elements that make up faces and possibly not that influential in the STS-FA. However, the following consideration speaks against the validity of this criticism. As already shown by Wollaston in the 19th century (Wollaston, 1824), estimates of eye gaze depend on concurrent information on the orientation of the face. And this latter information is available in the GFP. This was shown by Laube et al. (2011) who could establish that the influence of head or face orientation on perceived eye direction, first described by Wollaston, finds its correlate in changes of the BOLD signal in the GFP. On the other hand, previous fMRI work on face perception has suggested that one of the hallmarks of the STS-FA is a stark interest in the changing aspects of faces which, like changes in eye and face orientation, are important for gaze-following (Hoffman and Haxby, 2000; Lee et al., 2010). Hence, the fact that the GFP and the STS-FA are distinct, although both handling information on oriented faces and most probably also oriented eyes, clearly indicates different functional roles. On the other hand, the anatomic vicinity may suggest an exchange of pertinent information between the two. However, if the GFP handles information on averted faces, why does it not light up in the passive viewing experiment? The answer is that its activation is most probably contingent on the presence of an object serving as goal for the gaze and observer’s intention to follow gaze.

We found the maximum BOLD response to faces in the STS-FA rather than in the FFA or OFA as many other studies (Engell and Haxby, 2007). The reason is that, in our passive task to elicit maximal responses in the STS-FA, the set of face stimuli used was confined to pictures of emotionally neutral faces with averted eyes with the head straight, known to be less suitable for the FFA or OFA (Hoffman and Haxby, 2000; Narumoto et al., 2001). On the other hand, in most of the studies yielding stronger responses in the FFA or OFA, the emphasis was on faces exhibiting direct eye gaze, stimuli that seem to favor identity-processing.

In Pitcher et al. (2011), a face-selective area in the right pSTS was reported that responded three times more strongly to dynamic faces than to static faces. Hence, one may speculate that the current study using static stimuli underestimated the true size of the STS-FA and therefore failed to reveal an overlap between the GFP and the STS-FA. We cannot exclude the possibility that more powerful face stimuli might have expanded the activated areas with the consequence of some overlap to emerge. However, given the fact that the mean Talaraich coordinates of the pSTS patch center as given by Pitcher et al. (2011), (54, -38, 4), and the coordinates of the GFP in our study, (50, –64, 2), are separated by 26 mm Euclidean distance clearly supports the conclusion of largely noncongruent patches, at least when a static face localizer is used to map face-selective areas.

Nonhuman primates follow head gaze to establish joint attention. This behavior emerges very early during the development of the individual (Tomasello and Carpenter, 2005; Tomasello et al., 2007). According to Marciniak et al. (2015), it is characterized by key features that make human eye gaze-following reflexlike, namely swiftness and incomplete cognitive control. As described earlier, monkey head gaze-following activates a patch of cortex (the monkey GFP) whose location bilaterally in the posterior STS is reminiscent of the location of the human GFP. Also, the monkey GFP is anatomically distinct, not showing overlap with any of the face patches that can be activated by the passive vision of faces (Tsao et al., 2003, 2006). As a matter of fact, the spatial relationship of the monkey GFP with respect to the posterior face patch (PL) and the middle face patches (ML, MF) is reminiscent of the spatial relationship of the human GFP to the most posterior face-selective area (OFA) and the two more anterior ones (FFA, STS-FA). This lends further support to the notion of a close correspondence of the respective architectures. The major difference seems to be the ability of the human architecture to integrate social cues, providing directional information, other than head cues such as eye direction or the direction of fingers (Materna et al., 2008b; Laube et al., 2011). In other words, both species seem to exhibit a common core architecture for gaze-following, possibly reflecting homologous ancestry.

The notion of separate, yet possibly interdependent, cortical structures for the processing of faces and in particular faces showing gaze aversion and gaze-following is interesting with regard to observations on subjects with autism spectrum disorder (ASD). At least some people with ASD seem to be able to distinguish between different eye gaze positions when tested in discrimination tasks, suggesting an intact face-processing network. However, they fail to use information provided by the other’s face to follow the gaze and establish joint attention (Baron-Cohen, 1995; Leekam et al., 1998, 2000). In accordance with these behavioral observations, Pelphrey et al. (2005a) reported a lack of differentiation in the STS BOLD responses of ASD subjects when confronted with averted target-directed and averted non–target-directed eye gaze stimuli, a deficit that may reflect an inability to integrate information on the other’s gaze and the object of interest. The tentative conclusion suggested by these findings may be one of differential vulnerability of the face-processing network and the GFP, with the latter selectively compromised in ASD. However, what exactly is the added value of the GFP? At this stage, the lack of knowledge of the neuronal computations inside the GFP does not allow more than an admittedly rather vague speculation. We think that the GFP may be needed to convert directional information on eye and face/head orientation as well as directional information offered by other parts of the body into a “vector” describing the necessary shift of the observer’s “spotlight of attention” to the place of interest. Moreover, to ultimately lead to the establishment of joint attention devoted to an object found in a particular place, the GFP may also help to integrate information on the object at stake. Finally, to be viable, these calculations require the integration of knowledge on the observer’s viewpoint. A final remark pertains a possible role of the most anterior member of the face-processing network, the IFG-FA, located at the junction of inferior frontal sulcus and the precentral sulcus in gaze-following. There is evidence that the BOLD response of IFG-FA to faces is primarily driven by the eyes, i.e., the response to faces with eyes is lower than the presentation of the eyes alone and higher than to faces without eyes (Chan and Downing, 2011). In view of these findings and, moreover, the proximity of the IFG-FA to the frontal eye field, the authors speculated that it might contribute to analyze others’ gaze to elicit gaze-following movements of the observer. Hence, future work will have to address the possibility that not the face patch immediately neighboring the GFP but a much more remote anterior face patch, the IFG-FA, may serve as the major source of directional information provided by the eyes and the face.

## Synthesis

Reviewing Editor: Daniela Schiller, Mount Sinai School of Medicine Decisions are customarily a result of the Reviewing Editor and the peer reviewers coming together and discussing their recommendations until a consensus is reached. When revisions are invited, a fact-based synthesis statement explaining their decision and outlining what is needed to prepare a revision will be listed below. The following reviewer(s) agreed to reveal their identity: Galit Yovel, Rufin Vogels.

The revised manuscripts adequately addresses the previous concerns, but another issue remains:

Figure 8 presents the contrasts face vs no face and GF vs eye color matching in the STS face area and GF area. The large and significant response to face vs no face in the face area and the gaze vs color task in the GF area - are due to double dipping - it shows the response of the same data that was used to define these areas - and therefore overestimates the degree of face/gaze selectivity in these areas. You should therefore remove these contrasts and only report the contrasts face vs no face in the GF area and the gaze vs color in the face area - to demonstrate that these areas do not overlap at the level of the individual subject.
